# Considerations Regarding the Periprocedural Use of Oral Anticoagulation Therapy in Patients Undergoing Atrial Fibrillation Ablation

**DOI:** 10.19102/icrm.2018.090806

**Published:** 2018-08-15

**Authors:** James A. Reiffel

**Affiliations:** ^1^Electrophysiology Section, Division of Cardiology, Department of Medicine, Columbia University, New York, NY, USA

**Keywords:** Ablation, anticoagulation, atrial fibrillation

In this issue of *The Journal of Innovations in Cardiac Rhythm Management*, Maraj et al.^1^ report on the periprocedural use of oral anticoagulation therapy in patients undergoing atrial fibrillation (AF) ablation. This manuscript provides a useful review of the issues involved in and the therapeutic choices available with respect to, pre-, intra-, and postprocedural anticoagulant usage. Included is the correct view that we have learned from the earlier days of ablation as well as current approaches that have prompted a reduction in the risk of major bleeding complications while simultaneously keeping embolic consequences low.

Presently, it is widely known that (1) anticoagulation cannot be safety discontinued without prompting thromboembolic risk in a patient with AF and high-stroke-risk markers prior to intervention for any prolonged period of time (as determined according to the specific agent used and any pharmacodynamic and pharmacokinetic decisions involved in its dosing regimen); (2) anticoagulation is required during AF ablation, as it is during any left heart catheter-based procedure, to prevent procedure-related left heart clot formation and embolization; and (3) anticoagulation is recommended for continued long-term therapy in AF patients who demonstrate high-stroke-risk marker profiles (eg, CHA_2_DS_2_-VASc score ≥ 2) prior to ablation. It is this last point that sometimes goes unheeded in the hopes that ablation for AF will alleviate the risk of thromboembolism. However, though such a desire is reasonable, this goal is typically unattainable for one or more of several reasons. First, ablation is not uniformly successful, especially in patients with more advanced forms of AF and more severe associated structural disorders. This is true particularly if these patients are evaluated with prolonged monitoring techniques rather than just symptom assessment and intermittent electrocardiograms. AF recurrences are common postablation as time goes by, and “silent” AF presentations are more common with postablation monitoring than they are prior to ablation.^2^ Second, the resolution of AF, whether by ablation or by antiarrhythmic drug use, does not assure that the atrial pathophysiology consequent to the AF (morphologic, functional, endothelial, or biochemical) will necessarily resolve. Third, the concomitant disorders associated with the patient’s CHA_2_DS_2_-VASc score do not disappear with the ablation of AF, and these also confer an embolic risk—most likely to an even greater degree when combined with any residual left-atrial effects from the AF. Fourth, while some reports of postablation patients do suggest that anticoagulation may be safely discontinued, they have tended to involve relatively lower-risk patients with paroxysmal AF and/or relatively short periods of follow-up (rather than a period of five years or more).

Finally, and independent of the above, I want to take this opportunity to remind readers that if anticoagulation is being performed with a novel oral anticoagulant (NOAC) and the NOAC is to be withheld prior to ablation, then the ablationist must consider the patient’s renal function and concomitant drug treatments when determining how long it will take the NOAC effect to wash out. Otherwise, an unwanted anticoagulant effect might remain due to incomplete renal excretion or inhibited hepatic drug metabolism, potentially complicating the ablation procedure.

Accordingly, I hope that the readers of the paper by Maraj et al.^1^ will appreciate the useful insight it contains while simultaneously applying its information effectively and safely.
